# Diffusion MRI connectometry automatically reveals affected fiber pathways in individuals with chronic stroke^☆^

**DOI:** 10.1016/j.nicl.2013.06.014

**Published:** 2013-06-29

**Authors:** Fang-Cheng Yeh, Pei-Fang Tang, Wen-Yih Isaac Tseng

**Affiliations:** a Department of Biomedical Engineering, Carnegie Mellon University, PA, USA; b School and Graduate Institute of Physical Therapy, National Taiwan University College of Medicine, Taipei, Taiwan; c Center for Optoelectronic Medicine, National Taiwan University College of Medicine, Taipei, Taiwan; d Department of Medical Imaging, National Taiwan University Hospital, Taipei, Taiwan; e Graduate Institute of Brain and Mind Sciences, National Taiwan University College of Medicine, Taipei, Taiwan

**Keywords:** Diffusion MRI, Diffusion spectrum imaging, Structural connectivity, Spin distribution function, Connectometry

## Abstract

Building a human connectome database has recently attracted the attention of many researchers, although its application to individual subjects has yet to be explored. In this study, we acquired diffusion spectrum imaging of 90 subjects and showed that this dataset can be used as a norm to examine pathways with deviant connectivity in individuals. This analytical approach, termed diffusion MRI connectometry, was realized by reconstructing patient data to a common stereotaxic space and calculating the percentile rank of the diffusion quantities with respect to those of the norm. The affected tracks were constructed with deterministic tractography using the local tract orientations with substantially low percentile ranks as seeds. To demonstrate the performance of the connectometry, we applied it to 7 patients with chronic stroke and compared the results with lesions shown on T_2_-weighted images, apparent diffusion coefficient (ADC) maps, and fractional anisotropy (FA) maps, as well as clinical manifestations. The results showed that the affected tracks revealed by the connectometry corresponded well with the stroke lesions shown on T_2_-weighted images. Moreover, while the T_2_-weighted images, as well as the ADC and FA maps, showed only the stroke lesions, connectometry revealed entire affected tracks, a feature that is potentially useful for diagnostic or prognostic evaluation. This unique capability may provide personalized information regarding the structural connectivity underlying brain development, plasticity, or disease in each individual subject.

## Highlights

• Diffusion MRI connectometry can identify tracks with decreased connectivity.
• T_2_-weighted images, and ADC, and FA maps show only the stroke lesions.
• Diffusion MRI connectometry reveals the entire affected pathways.

## 1 Introduction

Cerebral connectivity is believed to play an important role in the function of the human brain and could aid in the discovery of disease biomarkers (Akil et al., 2011). Measuring the structural connectivity of the human brain in vivo is a major challenge in the field of neuroscience. By modeling diffusion MRI data with a diffusion ellipsoid, diffusion tensor imaging (DTI) (Basser et al., 1994) can characterize the structural integrity of axonal fibers via fractional anisotropy (FA) and apparent diffusion coefficient (ADC) analyses (Basser and Pierpaoli, 1996; Pierpaoli and Basser, 1996; Pierpaoli et al., 1996). The principle orientation of the diffusion tensor can also facilitate fiber tracking to reveal structural connectivity (Basser et al., 2000; Conturo et al., 1999; Mori et al., 1999). Although DTI has been widely used in group studies, its application to individual patients remains hampered, partly due to the limitation that the tensor model can only describe fibers with a single orientation and that it is subjected to various sources of partial volume effects (Alexander et al., 2001; Metzler-Baddeley et al., 2012; Oouchi et al., 2007).

More advanced diffusion MRI methods have been proposed to provide superior angular resolution and to overcome the limitations of DTI (Jones et al., 2012). Studies have used high angular resolution diffusion imaging (HARDI) (Tuch et al., 2002) and diffusion spectrum imaging (DSI) (Wedeen et al., 2005) to model the diffusion characteristics of axonal fibers. These methods provide orientation distribution functions of the diffusion to resolve multiple fiber orientations, which can be used in deterministic fiber tracking to delineate fiber trajectories (Hagmann et al., 2008; Honey et al., 2009; Wedeen et al., 2008). This progress further led to the study of the connectivity matrix (Hagmann et al., 2007; Hagmann et al., 2010a) and its application to real world research problems (Hagmann et al., 2010b; Robinson et al., 2010). Although connectivity analysis has been applied to group wise studies, its application to individual subject is yet to be explored.

In this paper, we acquired diffusion spectrum imaging of 90 subjects and used it as a norm to examine deviant connectivity in individuals with chronic stroke. Two novel strategies were adopted to carry out this analysis. First, the diffusion data was reconstructed in a common space using the q-space diffeomorphic reconstruction (QSDR) algorithm (Yeh and Tseng, 2011), which is a model-less reconstruction approach that applies spatial normalization to diffusion data. QSDR transforms the distribution of diffusion spins to a template space based on a given deformation field, and the transformed distribution can be used to calculate the spin distribution function (SDF), which quantifies the amount of spins that diffuses at any orientation. SDF is similar to diffusion orientation distribution function estimated using DSI, but the difference is that SDF scales with spin density, thus making it comparable across voxels and less susceptible to corrupted signals due to partial volume effect (Yeh et al., 2011). Moreover, SDF has an analytical solution, and it is less susceptible to error in numerical estimation (Yeh et al., 2010). The SDFs of the normal population can be used as a norm to obtain the percentile ranks of the diffusion quantities in an individual, and local tract orientations with a substantially low percentile rank indicated a potential connectivity change.

The second strategy is that instead of measuring cortical–cortical connectivity and comparing their differences, we first obtained the local differences in SDFs and then track *only* those with substantial differences to reveal the affected portion of the fiber pathways. This strategy bypassed the complexity of defining cortical–cortical connectivity and thus less affected by the limitation of fiber tracking algorithm in differentiating branching or crossing patterns. This analytical method, termed diffusion MRI connectometry, provides personalized information of the studied subject and constitutes a new diagnostic tool of brain disease.

To demonstrate the performance of diffusion MRI connectometry, we applied the connectometry to 7 patients with chronic stroke to examine their affected tracks. As the affected tracks should correspond to the chronic stroke lesions shown on T_2_-wieghted images, we used the findings on the T_2_-wieghted images to assess the accuracy of the connectometry.

## 2 Materials and methods

### 2.1 Subjects

A total of 90 healthy subjects and 7 patients with chronic stroke were recruited in the study. The study was approved by the Research Ethics Committee of the National Taiwan University Hospital, and written informed consent was obtained from the participants. The healthy subjects (45 males and 45 females) had no previous history of neurological or mental disorders. The mean ages of the male and female volunteers were 32.58 (standard deviation = 12.96) and 33.58 years of age (standard deviation = 12.26), respectively, and the age difference was not statistically significant (*p* = 0.7078, two-tail). All 7 patients had a first-episode stroke approximately 6 months prior to the study. The NIHSS score ranged from 2 to 8, equivalent to mild to moderate stroke (see Table 1 for the demographics).

### 2.2 MRI acquisitions

All subjects were scanned in a 3 T MRI scanner (Trio, Siemens, Erlangen, Germany) using a 12-channel phased-array head coil. All of the images were prescribed in a trans-axial view parallel to the anterior commissure–posterior commissure line. T_2_-weighted images were acquired with a fast spin echo sequence, TR/TE = 5920/102 ms, slice thickness = 3.0 mm, field of view = 25 × 25 cm, matrix size = 256 × 256, and slice number = 35. DSI was performed using a pulsed-gradient spin-echo diffusion EPI sequence with twice-refocused balanced echoes (Reese et al., 2003). The maximum diffusion sensitivity (b-max) was 6000 s/mm^2^ and TR/TE was 9100/142 ms. The in-plane spatial resolution was 2.9 mm, and the slice thickness was 2.9 mm.

A total of 203 grid sampling points were sampled in the diffusion-encoding space (q space), as was proposed in an optimization study (Kuo et al., 2008). Forty-five slices were acquired to cover the entire brain. The total scan time was approximately 45 min.

### 2.3 Reconstructing spin distribution functions in a common space

The diffusion data of each subject were reconstructed in a common stereotaxic space using q-space diffeomorphic reconstruction (QSDR) (Yeh and Tseng, 2011), a method that satisfies the conservation of diffusible spins and can be applied to diffusion datasets acquired with different diffusion sampling schemes, including the single shell scheme (also known as high angular resolution diffusion images, HARDI), multiple shell scheme, and grid scheme (also known as the DSI scheme). QSDR uses diffusion signals to calculate the spin distribution function (SDF), $\psi \left(r,\hat{u}\right)$, which is defined as the number of spins at location **r** that diffuse along the orientation $\hat{u}$. The formula for the calculation of SDF is as follows:

(1) $$\psi \left(r,\hat{u}\right)=\left|J_{\varphi }\left(r\right)\right|Z_{0}\sum_{i}W_{i}\left(\varphi \left(r\right)\right)\mathrm{sinc}\left(\sigma \sqrt{6Db_{i}}<{\hat{g}}_{i},\frac{J_{\varphi }\left(r\right)\hat{u}}{\left\|J_{\varphi }\left(r\right)\hat{u}\right\|}>\right)$$

where **r** represents the coordinates in the template space, and the function φ is a mapping function that maps a template space coordinates **r** to its corresponding coordinates in the subject's native space. The mapping function φ was obtained by registering the FA maps of the subject to the FMRIB 1 mm FA template (FSL, Oxford, UK) using a nonlinear registration (Ashburner and Friston, 1999) implemented in DSI Studio (http://dsi-studio.labsolver.org). The goodness-of-fit was evaluated using the R^2^ between the warped image and template image. All patients in this study had R^2^ greater than 0.64. *J*_*φ*_(**r**) is the Jacobian matrix of the mapping function at **r**, and |*J*_*φ*_(**r**)| is the Jacobian determinant. *W*_*i*_(*φ*(**r**)) is the diffusion MR signals at *φ*(**r**) and can be estimated numerically using trilinear interpolation. *b_i_* and ${\hat{g}}_{i}$ are the b-value and the diffusion gradient direction of the diffusion signal, respectively. *σ* is the diffusion sampling ratio, for which a recommended value of 1.25 was used in this study. *D* is the diffusivity of water. *Z*_0_ is the constant estimated by the diffusion signals of free water diffusion, as conducted in our original study (Yeh and Tseng, 2011). The free water calibration was automatically conducted using voxels located in the lateral ventricles at ( 6, 0, 18) and (− 6, 0, 18) in the Montreal Neurological Institute (MNI) space. The QSDR reconstruction yields maps of SDFs at 2 mm isotropic resolution. To estimate the number of anisotropic spins, each reconstructed SDF is subtracted by its minimum value. After the QSDR reconstruction and minimum subtraction, the SDF value can then be used to estimate the number of spins that diffuse preferentially along the fiber orientation. Our previous study demonstrated that the SDF value was linearly related to the volume fraction of the fibers regardless of the partial volume effect from the isotropic component (Yeh et al., 2010), thereby making it an index for measuring local structural connectivity.

### 2.4 Diffusion MRI connectometry

**Fig. 1:**
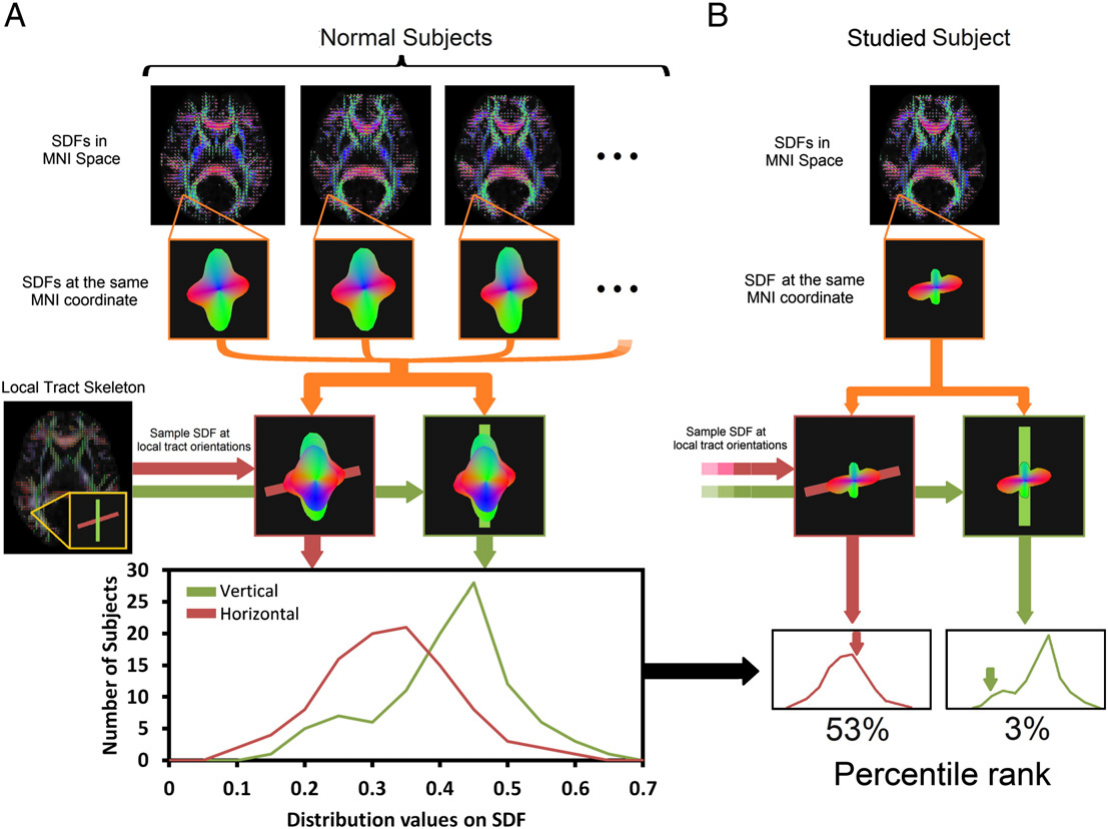
Flowchart of diffusion MRI connectometry. (A) The spin distribution functions (SDFs) were reconstructed in the MNI space using q-space diffeomorphic reconstruction. Then, for each voxel, the SDFs of the subjects were sampled in the local tract orientations to obtain the SDF value of the local tract orientation. A distinct distribution of the sampled SDF values was obtained for each local tract orientation. (B) The data of the studied subject were reconstructed in the MNI space, and the SDFs also received the same sampling procedure. The sampled SDF values were compared with the distribution of the normal population to obtain the percentile rank. The example here shows that the subject had a decreased SDF value in the vertical tract orientation, with the percentile rank dropping to the 3rd percentile.

To estimate the diffusion quantities along the local tract orientation, the SDFs were sampled using a “local tract skeleton”, as shown in Fig. 1A. This skeleton was obtained by averaging all of the subjects' SDFs and using the peaks of the averaged SDFs as the local tract orientations. Only the orientations in the white matter were selected. This skeleton is available for public access (http://dsi-studio.labsolver.org/download-images). One should note that a voxel may contain multiple local tract orientations, with each having a specific corresponding SDF value. As shown in Fig. 1A, the DSI data of the normal subjects were reconstructed by QSDR to obtain the SDFs in the MNI space. The SDF values at the same MNI coordinates were then sampled in each of the local tract orientations defined by the skeleton. Fig. 1A shows an example voxel with two local tract orientations (red and green sticks). By sampling the SDF value along the horizontal tract orientation (red stick) on each subject, we obtained the distribution of the sampled SDF values. The distribution of the SDF values in the vertical tract orientation (green stick) was also obtained in the same way. These two empirical distributions served as a norm to obtain the percentile rank of the SDF values of the studied subjects, as shown in Fig. 1B. The DSI data of a studied subject were also reconstructed by QSDR to obtain the SDFs in the MNI space. The same sampling procedure was applied to obtain the SDF values in the two local tract orientations. In this example, the SDF value in the horizontal tract orientation was close to normal with a percentile rank of 53%, whereas the value in the vertical tract orientation was decreased; the percentile rank dropped to 3%. The ranking procedure was conducted to all local tract orientations in the skeleton, and the percentile ranks were marked on each local tract orientation for each individual subject.

### 2.5 Constructing the affected tracks

**Fig. 2:**
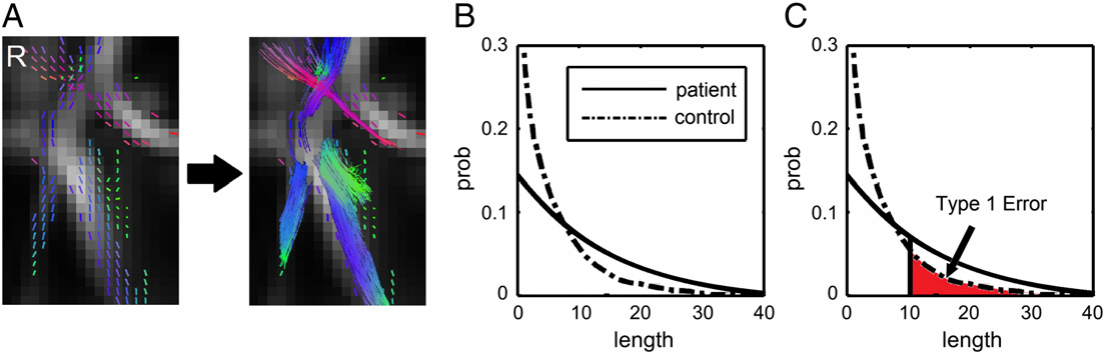
Construction of affected track and calculation of false discovery rate in diffusion MRI connectometry. (A) Local tract orientations with percentile ranks lower than a threshold are connected by a fiber tracking algorithm to reveal the affected tracks in patients. (B) The length distribution of the affected tracks follows an exponential distribution in normal population, whereas that in patients is shifted to the right. (C) The affected tracks with length greater than a length threshold (e.g. 10 voxel distance in this case) can be regarded as positive findings. The false discovery rate can be calculated by the area ratio between the two length distribution curves.

To show the segment of the tracks that were affected by the stroke, we performed fiber tracking on the local tract orientations with a percentile rank lower than 5, as shown in Fig. 2A, where local tract orientations with diffusion quantities (SDF values) lower than 5th percentile rank are plotted with directional colors (red: left–right, green: anterior–posterior, blue: superior–inferior), and tracks propagate along those local tract orientations. The tracking was conducted using a deterministic fiber tracking algorithm in DSI Studio. The tracking began from each local fiber orientation as seeds and propagated until no orientation was found in the propagation direction. A maximum turning angle of 60° was used with a step size of 1 mm (i.e., half of the spatial resolution in the template space). The obtained trajectories, termed the “affected tracks” hereafter, can be used to reveal the pathways with decreased connectivity. One should note that this fiber tracking, though computationally similar to conventional fiber tracking, is conceptually different because only those local tract orientations with substantial loss in diffusion quantities were tracked, and the affected tracks revealed only affected segments of the entire track pathways.

### 2.6 Length of affected tracks

The normal subject may also have affected tracks due to random variations, and we used the length of affected tracks (unit in voxel distance) to differentiate meaningful findings from random variations. The rationale behind it is that normal population may have “false-positive” affected tracks due to noise in diffusion weighted images, but its effect tends to appear randomly in space and results in fragments of affected tracks. As a result, the distribution of the length of the affected tracks (termed “length distribution” hereafter) will follow an exponential distribution since the concatenation of randomly distributed *local tract* orientations can be viewed as a Poisson process.

By contrast, the true connectivity difference in patient group will continue along fiber pathways and form longer affected tracks. The length distribution will thus be “shifted to the right” due to large amount of continuous tract orientations showing substantially lower ranks. Using length as an index, the difference between random effect and true connectivity difference can be differentiated and statistically tested. We can select the affected tracks with length greater than a threshold (termed “length threshold” hereafter) as positive findings because these trajectories are most likely more related to the disease. A higher length threshold may filter out most of the false discoveries (i.e., low Type 1 error) but may not be sensitive enough to subtle difference, whereas a lower length threshold may have better sensitivity but may include more false discoveries (i.e., high Type 1 error). The determination of the length threshold is a trade-off between sensitivity and specificity. To determine a reasonable value, we can quantify the Type 1 error of a length threshold using false discovery rate (FDR).

### 2.7 False discovery rate

The calculation of FDR requires the length distribution calculated from a group of subjects. In our study, we obtained the affected tracks of each patient by comparing his/her data with those of 90 normal subject, and the length of affected tracks in all patients were accounted to obtain the empirical length distributions for the patient group. Similarly, we obtained the affected tracks of each normal subject by comparing his/her data with those of the other 89 normal subjects to obtain the empirical length distribution for the normal population. An example of those empirical distributions is shown in Fig. 2B, where the length distribution of the patient group is shifted to the right due to increased length of affected tracks. As shown in Fig. 2C, the FDR of a length threshold is the ratio of the area under the distribution curves (the area is calculated from the threshold to infinity). A thought experiment is that if the distribution curve of the patient group is similar to that of normal group, then the area ratio is close to 1, meaning that almost all the findings are false discovery. This corresponds to FDR ≈ 1. Another example is that suppose we have the area of length > 20 being 0.0104 in the control group and 0.0491 in the stroke group, then given a total of 1000 affected tracks, the expected number of affected tracks with length greater than 20 is thus 0.0104 ∙ 1000 = 10.4 in the control group and 0.0491 ∙ 1000 = 49.1 in the stroke group. If we regard any affected track with length > 20 as stroke-related, the FDR is 10.4/49.1 = 0.2111, meaning that approximately one-fifth of the affected tracks are false discoveries. FDR provides a quantitative measurement of how sensitive/specific the finding is, and its value can also be used for risk assessment in clinical applications.

### 2.8 Public access to the analysis codes and local tract skeleton

The QSDR and related analysis used in this study were automatically conducted using DSI Studio. The analysis source codes are available for public access (http://dsi-studio.labsolver.org). To ensure the reproducibility of the results, we also provided the local tract skeleton on the website. A step-by-step guideline for obtaining diffusion MRI connectometry will be available in the online documentation.

## 3 Results

**Fig. 3:**
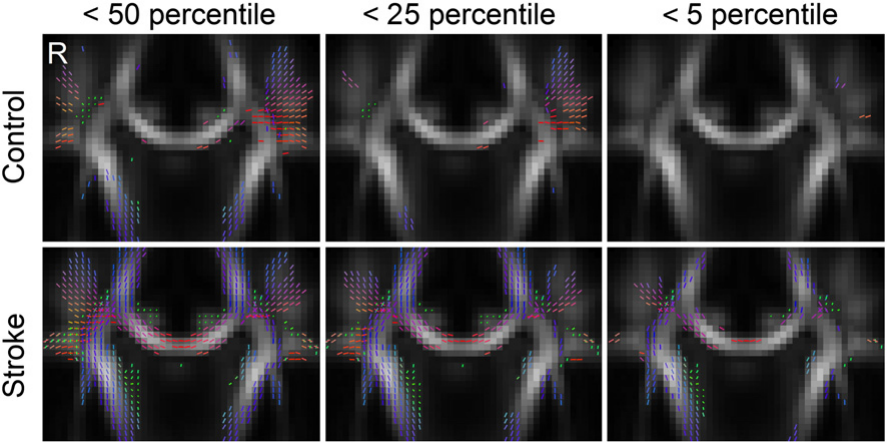
Diffusion MRI connectometry applied to a normal subject and to a patient with chronic stroke. The local tract orientations with diffusion quantities lower than 50th, 25th, and 5th percentile ranks were plotted with directional color coding. Both the control and the patient showed abundant local tract orientations with percentile ranks lower than 50, although very few local tract orientations with a percentile lower than 5 remained in the normal subject. The stroke patient displayed many fiber tracts affected by the stroke in the right internal capsule and right corpus callosum, where the local tract orientations were lower than the 5th percentile.

Fig. 3 shows the result of the percentile ranking analysis applied to a normal subject and a patient with chronic stroke. The local tract orientations with ranks lower than the 50th, 25th, and 5th percentiles are shown in coronal views with directional color coding (red: left–right, green: anterior–posterior, blue: superior–inferior). The analysis showed that both the stroke patient and normal subject had abundant local tract orientations with ranks less than the 50th percentile (i.e., below the median of the norm). By contrast, the normal subject had very few local tract orientations with less than a 5th percentile rank, whereas the stroke patient had abundant local tract orientations in the right corona radiata and right corpus callosum, indicating that these fiber pathways may have been affected by the stroke. We also accounted the percentage of local tract orientations with ranks less than 5th in both normal subject and patient groups. The normal subject group showed an average of 4.45% local tract orientations less than 5th percentage, a value that matches to the definition of 5th percentile rank. The discrepancy between 4.45% and 5% is due to discretization error (90 normal subject has a maximum discretization error of 1/90 = 1.11%). By contrast, the patient group showed an average of 17.18% local tract orientations less than 5th percentile rank, a value that increases substantially from the 5% baseline.

**Fig. 4:**
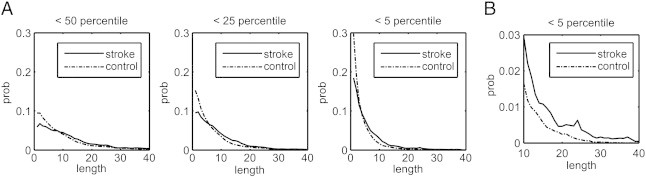
Length distribution of the affected tracks obtained from ranks lower than 50th, 25th, and 5th percentiles. (A) The length distribution in chronic stroke patients shows a shift to the right because many local tract orientations decreased the percentile ranks due to the stroke. (B) The magnification of the distribution at the 5th percentile threshold shows the probability of affected tracks with length greater than 30 in the patient group, whereas virtually none was seen in the control group. This suggests that the length threshold can be used to filter in fiber pathways with decreased connectivity due to the stroke episode.

Fig. 4A shows the length distributions of the affected tracks in 90 normal subjects and in 7 stroke patients. The distribution in normal subjects follows an exponential distribution, supporting our claim that the affected tracks in normal population are due to random effect. By contrast, we can see that patients' distributions are shifted to the right, suggesting that patients tend to have affected tracks with longer lengths. Fig. 4B shows the magnification of the length at percentile rank < 5, where the stroke group tends to have higher lengths than the control group. This result supports our claim that we can use a length threshold to differentiate meaningful finding from random effects.

**Fig. 5:**
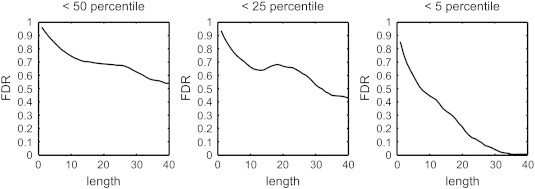
The false discovery rate of the length thresholds obtained from the affected tracks with ranks lower than the 50th, 25th, and 5th percentiles. The false discovery rate showed a decreasing trend as the length threshold increased, and this trend was most obvious at the 5% level. This result suggests that a higher length threshold can achieve a lower false discovery rate, allowing for us to identify fiber pathways with connectivity changes.

Fig. 5 shows the FDR at different length thresholds. Using the 5th percentile ranking, one can achieve a sufficiently low rate of false discovery at a higher length threshold. A length threshold of 10 had a false discovery rate of 0.4315, meaning that nearly half of the affected tracks with lengths greater than 10 may have been false positives. This false positive decreased to 0.2111 with lengths greater than 20 and to 0.0424 with lengths greater than 30. The analysis results suggest that we can control the length threshold to obtain the affected tracks with different rates of false discovery.

**Fig. 6:**
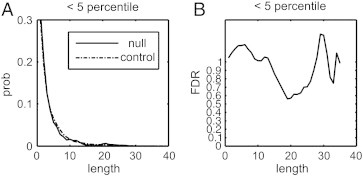
Results of a null experiment that selected 7 normal subjects as the patients. (A) The length distribution of the 7 subjects is plotted against that of normal distribution. The length distribution of the 7 subjects approximates that of the normal population, and there is no sign of right shifting. (B) The false discovery rate fluctuates around 1, suggesting that most of the findings are false positive.

Fig. 6 shows the result of a null experiment, which was conducted by randomly selected 7 normal subjects from our control group as hypothetical patients. The 7 subjects were selected using the randomization function provided by the C++ standard library. For each subject, the rank analysis was calculated with respect to the rest of 89 subjects, and the same fiber tracking algorithm was applied to obtain the affected tracks from local tract orientations with a rank lower than 5th percentile. Fig. 6A shows the empirical length distribution obtained from the 7 selected subjects plotted against that of normal subjects. The distribution of the 7 selected subjects approximates that of the normal populations and shows no “right shift”. The area ratio (FDR) also fluctuates around 1, as shown in Fig. 6B, suggesting that most findings are false discovery. Furthermore, the percentile analysis showed that only 3.62% of the local tract orientations in these 7 subjects had ranks less than 5th percentile. This value is substantially less than that (17.18%) in the stroke patient group. This null experiment demonstrates the specificity of our connectometry analysis.

**Fig. 7:**
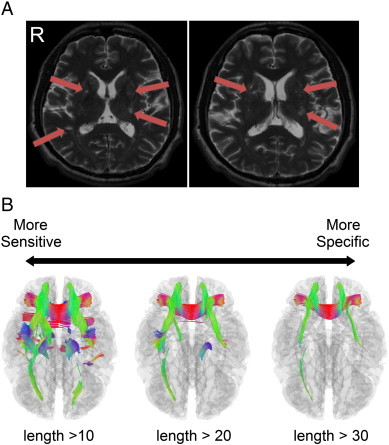
The results of the connectometry under different length thresholds. (A) An example of a subject (patient 2) with chronic stroke. The old infarct and lacunar lesions can be observed on the T_2_-wieghted images (arrows). (B) The corresponding connectometry of the subject at length thresholds of 10, 20, and 30 (unit in voxel distance). A higher length threshold provides more specific results to confirm the affected tracks, although it may fail to identify all of the possible affected tracks. By contrast, a lower length threshold is more sensitive to potentially affected tracks, although it may include more false positive results due to a higher false discovery rate. The optimal setting depends on the balance between sensitivity and specificity.

An example of different length thresholds applied to a patient with chronic stroke is shown in Fig. 7, whereas Fig. 7A shows the lesions on T_2_-weighted images, and Fig. 7B shows connectometry results with different length thresholds. A lower length threshold (e.g., 10) produces an exhaustive result of discoveries, but some affected tracks may not have a corresponding lesion on T_2_-weighted images. Alternatively, a higher length threshold (e.g., 30) may present with a more specific discovery, although some of the trajectories affected by certain lesions on T_2_-weighted images may be filtered out. The optimal setting depends on the application in the clinical setting. A confirmation examination may require a higher length threshold, whereas an exploration examination may use a lower length threshold.

**Fig. 8:**
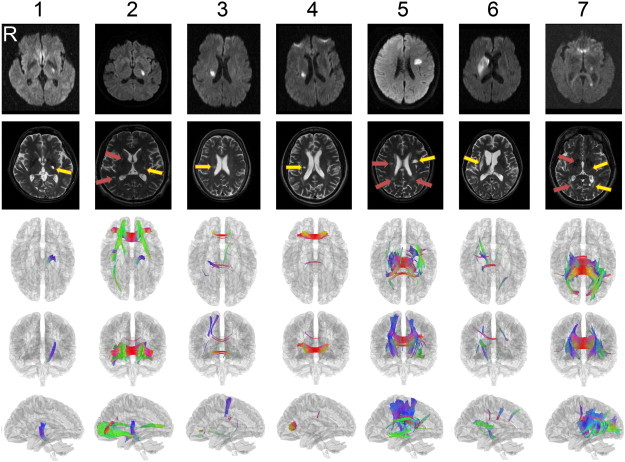
The results of the connectometry of the 7 patients with chronic stroke. The diffusion-weighted images acquired at the onset are shown in the first row, whereas the T_2_-wighted images acquired after 6 months are shown in the second row. The following rows show the connectometry in axial, coronal, and sagittal views. The affected tracks shown by the connectometry match the lesion sites shown on the diffusion weighted images and T_2_-wighted images, confirming the accuracy of the connectometry analysis. Despite old infarcts (yellow arrow) shown in the T_2_-wighted images, there are variable extents of old lacunar lesions (red arrows) in bilateral basal ganglia or periventricular white matter (arrows in patients 2, 5 and 7). Please refer to the texts and supplementary materials for a detailed description of each patient.

Fig. 8 shows the connectometry results of the 7 patients in addition to diffusion-weighted images and the T_2_-weighted images. The first row shows the diffusion-weighted images acquired at the onset of stroke, while the second row shows the T_2_-weighted images acquired approximately 6 months after onset. The following rows show the connectometry in axial, coronal, and sagittal views. The connectometry was calculated from DSI scans, which were acquired at the same time as the T_2_-weighted images. The affected tracks were constructed based on a 5th percentile threshold and a minimum length of 20. The diffusion weighted images, T_2_-weighted images and connectometry were reviewed by an experienced radiologist (W.Y.I.T.).

**Fig. S1:**
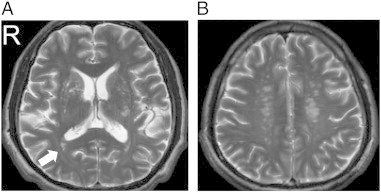
Patient 2 (49/M). (A) An old lacunar infarct in right temporal PVWM (arrow) and multiple old lacunar lesions in bilateral basal ganglia and thalami, and (B) multiple old lacunar infarcts in bilateral PVWM.

**Fig. S2:**
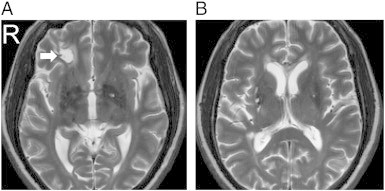
Patient 4 (51/M). (A) An old insult in right inferior frontal region (arrow), and (B) multiple old lacunar infarcts in bilateral basal ganglia.

**Fig. S3:**
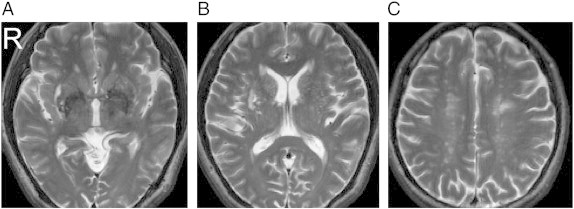
Patient 5 (51/M). (A) Multiple old lacunar infarcts in bilateral basal ganglia and right PVWM, (B) multiple old lacunar infarcts in bilateral basal ganglia, and (C) leukoaraiosis in bilateral PVWM.

**Fig. S4:**
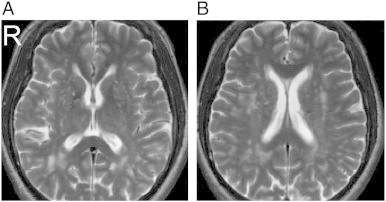
Patient 7 (53/M). (A) Multiple old lacunar infarcts in bilateral basal ganglia and PVWM, and (B) leukoaraiosis in bilateral PVWM.

The complete MRI findings are summarized in Table 2. One should note that due to the limitation of the figure, we can show only one T_2_-weighted image per subject. Lesions at other T_2_-weighted images are shown in Supplementary Figs. 1, 2, 3, and 4. A full report of the findings is listed in Inline Supplementary Table S1.

The complete MRI findings are summarized in Table 2. One should note that due to the limitation of the figure, we can show only one T_2_-weighted image per subject. Lesions at other T_2_-weighted images are shown in Supplementary Figs. 1, 2, 3, and 4. A full report of the findings is listed in Inline Supplementary Table S1.

Inline Supplementary Table S1

**Table S1:** Full report of findings

Patient 1 (55/M) has a stroke lesion in the left posterior limb of internal capsule (PLIC), and an old lacunar infarct in the left caudate head. The connectometry shows projection fibers (probably CST) from the stroke lesion down to the midbrain, but no fiber tracts arising from the lacunar lesion in the left caudate head.
Patient 2 (49/M) has a stroke lesion in the left PLIC and an old lacunar infarct in the right temporal periventricular white matter (PVWM). In addition, there are multiple old lacunar infarcts in bilateral basal ganglia, thalami and PVWM (Fig. S1). The connectometry shows projection fibers (probably CST) from the stroke lesion down to the midbrain, and association fibers arising from the old lacunar infarct in the right temporal PVWM to the right occipital cortex. In addition, there are multiple callosal fibers to bilateral frontal regions and multiple association fibers from bilateral medial temporal regions to medial prefrontal cortex.
Patient 3 (69/F) has a stroke lesion in the right PVWM, and leukoaraiosis in bilateral PVWM. The connectometry shows projection fibers and callosal fibers from the stroke lesion up to the right somatosensory cortex. There are also a few projection fibers from the left inferior frontal cortex down to the midbrain and a few callosal fibers in genu.
Patient 4 (51/M) has a stroke lesion in the right PVWM and an old insult in the right inferior frontal region (Fig. S2). In addition, there are multiple old lacunar infarcts in bilateral basal ganglia (Fig. S2). The connectometry shows no fiber tracts passing through the stroke lesion in the right basal ganglion, but prominent callosal fibers from the old insult to the left hemisphere. There is a small bundle of callosal fibers in the middle part of corpus callosum, which seems unrelated to the lesions.
Patient 5 (51/M) has a stroke lesion in the left posterior frontal white matter. There are multiple old lacunar infarcts in bilateral basal ganglia and the right PVWM, and leukoaraiosis in bilateral PVWM (Fig. S3). The connectometry shows projection fibers from the stroke lesion up to the left frontal cortex and down to the thalamus. There are multiple fibers in corona radiata and corpus callosum to bilateral fronto-parietal cortex, and association fibers including left arcuate fasciculus, left inferior fronto-occipital fasciculus and bilateral cingulum bundles.
Patient 6 (66/F) has a stroke lesion in the right basal ganglion. The connectometry shows projection fibers from the stroke lesion down to the midbrain. There are several small bundles of callosal fibers to the right fronto-parietal cortex and the left parietal cortex which seems not related to the stroke lesion.
Patient 7 (53/M) has several stroke lesions in the left thalamus, medial temporal and medial occipital regions. There are multiple old lacunar infarcts in bilateral basal ganglia and PVWM, and leukoaraiosis in bilateral PVWM (Fig. S4). The connectometry shows association fibers arising from the stroke lesions to the left occipital cortex, and callosal fibers to bilateral occipital–temporal cortex. There are multiple fibers in bilateral corona radiata extending from basal ganglia to PVWM and the left cingulum bundle.

Inline Supplementary Table S1

Inline Supplementary Table S1 can be found online at http://dx.doi.org/10.1016/j.nicl.2013.06.014.

As shown in Fig. 8 and summarized in Table 2, patients 1 and 2 had a stroke lesion in the left posterior limb of the internal capsule (PLIC). Both patients exhibited a segment of corticospinal tract (CST) with reduced connectivity from the stroke lesion down to the midbrain. In addition to the episode-related stroke lesions, patient 2 had multiple old lacunar strokes (also shown in Fig. 7) in bilateral basal ganglia (BG) and periventricular white matter (PVWM).

These correspond to bilateral association tracks and even the callosal tracks shown by connectometry. According to the patient demographics in Table 1, both patients suffered from severe motor weakness of the right upper extremities on day 30 (D30); the clinical manifestation might correspond to the affected CST segments found in the connectometry. The patients exhibited different degrees of motor recovery on day 180 (D180). The Fugl-Meyer upper extremity (FM UE) motor scores for patient 1 improved from 34 to 56, while patient 2 improved from 46 to 55. Patient 3 and patient 4 had a stroke lesion in the right PVWM. The connectometry in patient 3 showed a few projections as well as callosal tracks from the stroke lesion to the parietal cortex. In patient 4, the connectometry showed few tracks affected by the stroke lesion. Both patients had an equal FM UE motor score of 61 at D30 and comparable recovery at D180 (66 for patient 3 and 64 for patient 4). Patient 5 also had a stroke lesion in the left posterior frontal WM. He also presented with multiple old lacunar infarcts bilaterally in the basal ganglia and the right PVWM, as well as leukoaraiosis bilaterally in the PVWM. The connectometry showed that affected tracks extended from the stroke lesion, reaching the left frontal cortex (upward) and the thalamus (downward). In addition, connectometry shows affected tracks in the corona radiata and corpus callosum to the bilateral fronto-parietal cortex as well as in the association fibers, including the left arcuate fasciculus, left inferior fronto-occipital fasciculus and the cingulum bundles bilaterally. Although this patient had widespread lesions in white matter, connectometry shows that the affected tracks are distant from the CST. This might correspond to his FM UE motor scores, which were comparable to those in patient 3 and patient 4, who both had relatively high levels of motor functions. Patient 6 had a single stroke lesion in the right basal ganglion, whereas patient 7 had multiple stroke lesions in the left thalamus as well as in the medial temporal and medial occipital regions. Patient 7 also had multiple old lacunar infarcts in bilateral basal ganglia and PVWM, as well as leukoaraiosis in the PVWM bilaterally. The connectometry of patient 6 showed affected tracks projected from the stroke lesion down to the midbrain, whereas patient 7 showed association tracks arising from the stroke lesions to the left occipital cortex, as well as callosal tracks to bilateral occipital–temporal cortex. In addition, patient 7 showed multiple affected tracks in bilateral corona radiata extending from the basal ganglia to the PVWM and the left cingulum bundle. Although they presented with drastically different extents of stroke lesions and affected tracks, both patients had comparable FM UE scores at D30 (64 for patient 6, 63 for patient 7) and D180 (66 for patient 6, 66 for patient 7).

**Fig. 9:**
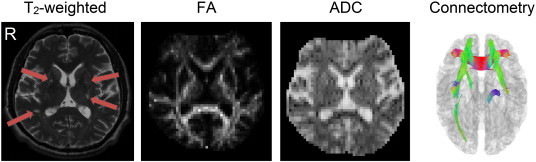
Diffusion MRI connectometry compared with T_2_-weighted images, as well as the ADC and FA maps in patient 2. The T_2_-weighted image shows higher signal intensity at the lesion locations, whereas the ADC map shows a subtle increase and the FA map shows a subtle decrease in the lesion site. None of these three modalities was able to reveal the entirety of the affected tracks. By contrast, connectometry presented the affected tracks that matched the lesion locations.

Fig. 9 compares the connectometry with the T_2_-weighted image, the apparent diffusion coefficient (ADC) map, and the FA map using data from patient 2. The T_2_-weighted image shows distinct high-intensity patches in the lesions, whereas the ADC and FA calculated from the DSI dataset show very subtle differences, which offer limited diagnostic or prognostic values. By contrast, the connectometry, which was calculated from the same dataset, shows the entirety of the affected tracks that enhance the conspicuity of the regions affected by the stroke. The locations and extents of the affected tracks revealed by the connectometry cannot be predicted based only on the T_2_-weighted images, or on the ADC or FA maps.

**Fig. 10:**
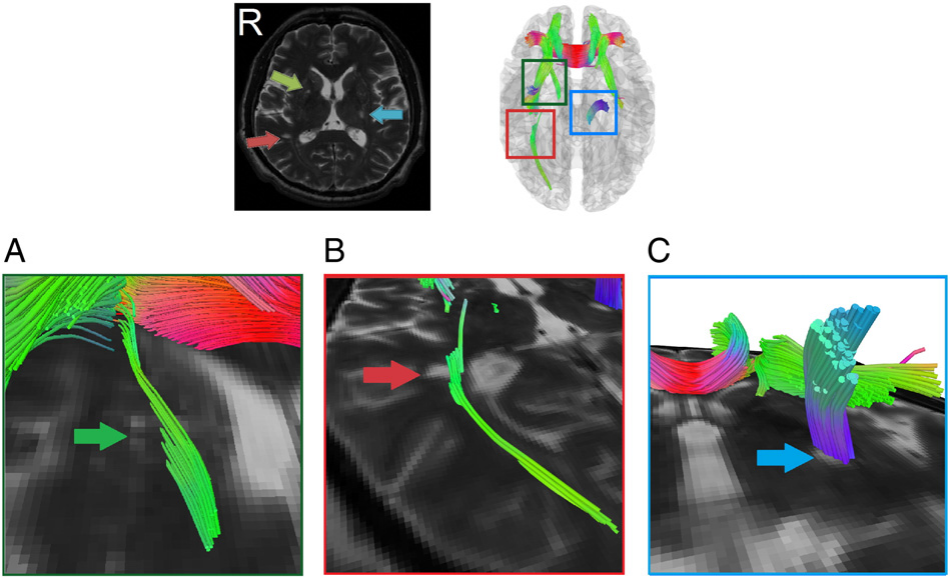
Connectometry results overlaid with T_2_-weighted images to demonstrate its localization power. Although connectometry was calculated from DSI data that were acquired at a relatively low resolution (2.9 × 2.9 × 2.9 mm^3^), its localization power was comparable to the spatial resolution of the T_2_-weighted images (1 × 1 × 3 mm^3^). The magnification of images further present how closely the affected tracks co-localized with the lesions shown on the T_2_-weighted images.

Fig. 10 further demonstrates the localization power of the connectometry using data from patient 2. The figure shows an old lacunar infarct in the right basal ganglia (green arrow), an old lacunar infarct in the right temporal PVWM (red arrow), and a stroke lesion in the left PLIC (blue arrow). The connectometry in Fig. 10A shows affected tracks arising from the old lacunar infarct in the right basal ganglia to the right frontal cortex, matching the lesion indicated by the green arrow. Similarly, the connectometry in Fig. 10B shows affected tracks coming from the right PVWM the right occipital cortex. The connectometry precisely matches the lesion site. Furthermore, the connectometry in Fig. 10C shows the affected tracks from the stroke lesion to the midbrain, with the fibers matching well with the stroke lesion shown on the T_2_-weighted image. Although the connectometry was calculated using DSI data, which were acquired at a relatively low resolution (2.9 × 2.9 × 2.9 mm^3^), the connectometry appears to be able to locate the stroke lesions at a resolution higher than the resolution of its original data.

## 4 Discussion

We developed an automatic analytical method referred to as diffusion MRI connectometry to identify fiber pathways with deviant connectivity in each individual subject. We also demonstrated its performance in patients with chronic stroke. This method was realized by first obtaining the empirical distribution of the SDF values from a normal population. The empirical distribution was then used as a norm to calculate the percentile ranks of the SDF values for individual patients. By tracking local tract orientations with a percentile rank lower than a predefined threshold, we constructed affected tracks with reduced connectivity in each individual patient. While the T_2_-wieghted images as well as the ADC and FA maps only showed the lesion sites, the connectometry revealed the entirety of the affected tracks as well as the pathways associated with the stroke lesions. The affected tracks varied between patients depending on the locations and extents of the stroke lesions and old lacunar strokes. These affected tracks also impaired motor function when they involved the CST.

To the best of our knowledge, diffusion MRI connectometry is the first diffusion MRI analysis method that examines connectivity changes on individual subjects using a norm of a normal population dataset. Current diffusion MRI analysis methods (Hua et al., 2008; Kanaan et al., 2006; Pagani et al., 2005) have mainly focused on group-level analysis and thus cannot be used to study connectivity changes at the individual level. The success of the connectometry lies in the combination of the fiber-specific SDF value and the norm of a normal population dataset. We have previously shown that the SDF value in a specific direction represents the volume fraction of fiber tracts in that direction and can be used to indicate the local structural connectivity in that particular direction (Yeh et al., 2010). This fiber-specific index cannot be replaced by an index that loses fiber specificity, such as FA, or by an orientation distribution function (ODF) value that cannot be compared across voxels. The connectometry also requires a template of a normal population to provide the local tract skeleton and the distribution of the SDF values corresponding to a specific local tract orientation. By combining these two essential components, connectometry allowed us to identify local tract orientations with SDFs that were deviant from the norm of the SDF values. We then constructed the affected tracks from the identified local tract orientations. In this study, we have successfully applied this novel method to patients with chronic stroke and demonstrated its capacity to reveal affected tracks in each individual patient.

Diffusion MR connectometry can provide spatial resolution beyond the original acquisition parameters of the diffusion MRI. In this study, the diffusion MRI was acquired at an isotropic spatial resolution of 2.9 mm^3^, whereas the T_2_-weighted images were acquired at an in-plane resolution of 1 mm. Despite the resolution gap, the affected tracks identified by the connectometry still co-localized with the spatial locations of the stroke lesions shown on the T_2_-weighted images. This surprising finding indicates that low-resolution diffusion MRI does not compromise the accuracy of the connectometry in lesion localization, supporting the recent notion that diffusion tractography can achieve a resolution beyond that of the original diffusion-weighted images (Calamante et al., 2010).

From the patient study, we know that the affected tracks shown in the connectometry resulted either from episode-related stroke lesions or from old lacunar infarcts/insults. There were certain tracks that appeared to be unrelated to both causes. These fibers constituted a minority of the total affected tracks and may have accounted for 20% of FDR at a length threshold of 20. The motor function was predominantly determined by the location of the stroke lesion (PLIC) and the accompanying CST damage. If the stroke lesion was distant from the CST course, the motor impairment was rather mild, regardless of the locations and extents of the affected tracks. It is interesting that 4 out of the 7 patients had multiple lacunar infarcts in bilateral basal ganglia and periventricular white matter. These 4 patients exhibited variable locations and extents of the affected tracks. In addition to sensorimotor dysfunction, these patients may have been inflicted by variable degrees of cognitive impairment (De Reuck and Van Maele, 2009; Jacova et al., 2012). Given that the locations and extents of the affected tracks vary from patient to patient, connectometry may be helpful in identifying patients who are at risk of vascular dementia. Further studies with large sample sizes are required to clarify the relationships between affected tracks and clinical manifestations.

Moreover, our study showed that connectometry is more sensitive than FA or ADC mapping. While the affected tracks could not be identified from either FA or ADC mapping, connectometry showed affected tracks extended from the lesions shown in T_2_-wighted images. The difference in sensitivity can be attributed to the fact that tensor analysis is often affected by a variety of partial volume effects and that the results are often blended with those due to CSF contamination or crossing fibers, making it hard to differentiate the causes. The SDFs provided by QSDR is orientation sensitive, and the CSF contamination problem can be handled by spin density information (Yeh et al., 2011), thus offering a clearer picture of affected tracks.

Connectometry has several features that are worth mentioning. First, the connectometry algorithm is fully automatic. Because it requires no manual operation, such as ROI selection or manual editing, it is not subject to human intervention. This feature also allows for the fast screening of brain diseases. Second, connectometry allows for the determination of the false discovery rate of the affected tracks. This feature allows for users to adjust sensitivity and specificity according to the study purpose. For confirmatory evaluation, users may set up a stricter false discovery rate, whereas for exploratory purposes, users may allow for a higher false discovery rate to search possible affected tracks. Third, given the versatility of QSDR, connectometry is not restricted to one particular type of diffusion acquisition scheme. Although not demonstrated in this study, it is possible to use diffusion MR data acquired from a DTI scheme, single-shell scheme (e.g., HARDI), grid scheme (e.g., DSI) or other acquisition schemes. Moreover, connectometry is compatible with other HARDI or DSI templates, and its accuracy will likely improve as the quality of the templates also improves.

Current diffusion MRI connectometry also has certain limitations. Connectometry analysis is more sensitive to long-range fiber pathways than to short-range fiber pathways. This drawback is nonetheless understandable because long-range fiber pathways may affect the SDF values across several voxels, indicating that detecting changes in them will be easier than detecting differences in short-range fibers. One improvement to counter this drawback and to increase the detection power of connectometry would be to choose normal subjects that match well with the study patients (e.g., sex, age, handedness). The normal subjects recruited in this study are Asian, and whether the data can be applied to other ethnicity groups (e.g. Western/Caucasian) is questionable. A better patient–control match may reduce physiological differences and potentially increase the sensitivity of connectometry. Further works are required to investigate the differences in population-based templates across different age, gender or ethnic groups. The second drawback of connectometry is that a large number of normal subjects are needed to obtain a reliable percentile rank. For example, to achieve a minimum possible rank of 5th percentile for an individual, at least 20 matched normal subjects are needed, with even more subjects needed to avoid discrete errors in calculation. Lastly, connectometry is susceptible to EPI distortion in the frontal lobe (the location may depend on the phase encoding direction) and also the T_2_ shine-through effect. Nonetheless, there are several solutions available. QSDR can be combined with EPI distortion correction. The T_2_ shine-through can be corrected by measuring the T_2_ effect using different echo times. The lengthy scanning time had once been a problem, but this can be handled by new diffusion pulse sequences (Feinberg et al., 2010; Moeller et al., 2010; Porter and Heidemann, 2009; Reese et al., 2009). With the advance of novel image acquisition methods, it may be foreseen in the near future that a high angular resolution diffusion scan can be obtained with very minimal distortion and short scanning time.

In conclusion, diffusion MRI connectometry can identify fiber pathways with decreased connectivity in individual patients with chronic stroke. It offers a fully automatic analysis and is readily applicable to patients who suffer from various brain diseases. Future studies could use connectometry as a screening tool to automatically detect subclinical patients as well as to monitor the evolution of targeted pathways over time. Furthermore, by characterizing the spatial patterns of altered connectivity, connectometry can potentially reveal structural counterparts underlying the course of brain development, plasticity, and disease, thereby leading to the discovery of biomarkers of neurological or psychiatric disorders.

The following are the supplementary data related to this article.

Supplementary data to this article can be found online at http://dx.doi.org/10.1016/j.nicl.2013.06.014.

## Figures and Tables

**Table 1:** Patient demographics.

Patient	Age/sex	Stroke location in MRI	NIHSS	FM UE Motor at day 30	FM UE Motor at day 180
1	55/M	Left PLIC	7	34	56
2	49/M	Left PLIC	8	46	55
3	69/F	Right PVWM	5	61	66
4	51/M	Right PVWM	2	61	64
5	51/M	Left posterior frontal WM	3	61	66
6	66/F	Right BG	5	64	66
7	53/M	1. Left thalamus	7		
		2. Left medial temporal region		63	66
		3. Left medial occipital region			

BG: basal ganglia, FM: Fugl-Meyer, PLIC: posterior limb of internal capsule, PVWM: periventricular white matter.

**Table 2:** Summary of MRI findings and connectometry results.

Patient	Age/sex	Stroke location in MRI	Other findings in MRI	Connectometry related to stroke lesion(s)	Connectometry unrelated to stroke lesion(s)
1	55/M	Left PLIC	An old lacunar infarct in left caudate head	A CST segment from stroke lesion down to midbrain	None
2	49/M	Left PLIC	1. Multiple old lacunar infarcts in bilateral BG, thalami and PVWM 2. An old lacunar infarct in right temporal PVWM	A CST segment from stroke lesion down to midbrain	1. CC to bilateral frontal regions 2. Association fibers from bilateral medial temporal regions to medial prefrontal cortex 3. Association fibers from the old lacunar infarct to right occipital cortex
3	69/F	Right PVWM	Leukoaraiosis in bilateral PVWM	1. Projection fibers from stroke lesion up to right parietal cortex 2. CC from stroke lesion up to right parietal cortex	1. Projection fibers from left inferior prefrontal down to midbrain 2. CC in genu
4	51/M	Right PVWM	1. An old insult in right inferior frontal region 2. Multiple old lacunar infarcts in bilateral BG	None	1. CC from the old insult to left medial hemisphere 2. CC to left medial hemisphere
5	51/M	Left posterior frontal WM	1. Leukoaraiosis in bilateral PVWM 2. Multiple old lacunar infarcts in bilateral BG and right PVWM	1. Projection fibers from stroke lesion up to left frontal cortex and down to thalamus 2. Left AF and IFOF	1. Bilateral CR and CC to fronto-parietal cortex 2. Bilateral CB
6	66/F	Right BG	None	Projection fibers from stroke lesion down to mid brain	CC to right fronto-parietal cortex and left parietal cortex
7	53/M	1. Left thalamus 2. Left medial temporal region 3. Left medial occipital region	1. Leukoaraiosis in bilateral PVWM 2. Multiple old lacunar infarcts in bilateral BG and PVWM	1. Association fibers from stroke lesions to left occipital cortex 2. CC to bilateral occipito-temporal cortex	1. Bilateral CR from BG up to PVWM 2. Left CB

AF: arcuate fasciculus, BG: basal ganglia, CB: cingulum bundle, CC: corpus callosum, CR: corona radiata, IFOF: inferior fronto-occipital fasciculus, PLIC: posterior limb of internal capsule, PVWM: periventricular white matter, SM: sensorimotor.
