# Supplementary material for: Diffusion MRI connectometry automatically reveals affected fiber pathways in individuals with chronic stroke
Source: Neuroimage Clin. 2013 Jun 29;2:912–21. doi: 10.1016/j.nicl.2013.06.014 (PMC3777702; doi:10.1016/j.nicl.2013.06.014)
Supplement: Inline Supplementary Table S1 [file mmc1.docx]

| **Supplementary Table 1: MRI Findings** |
| --- |
| Patient 1 (55/M) has a stroke lesion in left posterior limb of internal capsule (PLIC), and an old lacunar infarct in left caudate head. The connectometry shows projection fibers (probably CST) from the stroke lesion down to the midbrain, but no fiber tracts arising from the lacunar lesion in left caudate head. |
| Patient 2 (49/M) has a stroke lesion in left PLIC and an old lacunar infarct in right temporal periventricular white matter (PVWM). In addition, there are multiple old lacunar infarcts in bilateral basal ganglia, thalami and PVWM (Fig. S1). The connectometry shows projection fibers (probably CST) from the stroke lesion down to the midbrain, and association fibers arising from the old lacunar infarct in right temporal PVWM to right occipital cortex. In addition, there are multiple callosal fibers to bilateral frontal regions and multiple association fibers from bilateral medial temporal regions to medial prefrontal cortex. |
| Patient 3 (69/F) has a stroke lesion in right PVWM, and leukoaraiosis in bilateral PVWM. The connectometry shows projection fibers and callosal fibers from the stroke lesion up to the right somatosensory cortex. There are also a few projection fibers from left inferior frontal cortex down to the midbrain and a few callosal fibers in genu. |
| Patient 4 (51/M) has a stroke lesion in right PVWM and an old insult in right inferior frontal region (Fig. S2). In addition, there are multiple old lacunar infarcts in bilateral basal ganglia (Fig. S2). The connectometry shows no fiber tracts passing through the stroke lesion in right basal ganglion, but prominent callosal fibers from the old insult to the left hemisphere. There is a small bundle of callosal fibers in the middle part of corpus callosum, which seems unrelated to the lesions. |
| Patient 5 (51/M) has a stroke lesion in left posterior frontal white matter. There are multiple old lacunar infarcts in bilateral basal ganglia and right PVWM, and leukoaraiosis in bilateral PVWM (Fig. S3). The connectometry shows projection fibers from the stroke lesion up to the left frontal cortex and down to the thalamus. There are multiple fibers in corona radiata and corpus callosum to bilateral fronto-parietal cortex, and association fibers including left arcuate fasciculus, left inferior fronto-occipital fasciculus and bilateral cingulum bundles. |
| Patient 6 (66/F) has a stroke lesion in right basal ganglion. The connectometry shows projection fibers from the stroke lesion down to the midbrain. There are several small bundles of callosal fibers to right fronto-parietal cortex and left parietal cortex which seems not related to the stroke lesion. |
| Patient 7 (53/M) has several stroke lesions in left thalamus, medial temporal and medial occipital regions. There are multiple old lacunar infarcts in bilateral basal ganglia and PVWM, and leukoaraiosis in bilateral PVWM (Fig. S4). The connectometry shows association fibers arising from the stroke lesions to left occipital cortex, and callosal fibers to bilateral occipital-temporal cortex. There are multiple fibers in bilateral corona radiata extending from basal ganglia to PVWM and left cingulum bundle. |
